# Procalcitonin Is a Prognostic Marker of Hospital Outcomes in Patients with Critical Limb Ischemia and Diabetic Foot Infection

**DOI:** 10.1155/2019/4312737

**Published:** 2019-08-14

**Authors:** Marco Meloni, Valentina Izzo, Laura Giurato, Enrico Brocco, Michele Ferrannini, Roberto Gandini, Luigi Uccioli

**Affiliations:** ^1^Diabetic Foot Unit, Department of Systems Medicine, University of Rome “Tor Vergata”, Viale Oxford 81, 00133 Rome, Italy; ^2^Diabetic Foot Unit, Foot and Ankle Clinic, Abano Terme Polyclinic, Piazza Cristoforo Colombo 1, 35031, Abano Terme, Padua, Italy; ^3^Division of Hypertension and Nephrology, Department of Systems Medicine, University of Rome “Tor Vergata”, Viale Oxford 81, 00133 Rome, Italy; ^4^Department of Interventional Radiology, University of Rome “Tor Vergata”, Viale Oxford 81, 00133 Rome, Italy

## Abstract

**Aim:**

To evaluate the prognostic role of procalcitonin (PCT) in patients with diabetic foot infection (DFI) and critical limb ischemia (CLI).

**Materials and Methods:**

The study group was composed of diabetic patients with DFI and CLI. All patients were treated according to a preset limb salvage protocol which includes revascularization, wound debridement, antibiotic therapy, and offloading. Inflammatory markers, including PCT, were evaluated at admission. Only positive values of PCT, greater than 0.5 ng/ml, were considered. Hospital outcomes were categorized as limb salvage (discharge with preserved limb), major amputation (amputation above the ankle), and mortality.

**Results:**

Eighty-six patients were included. The mean age was 67.3 ± 11.4 years, 80.7% were male, 95.1% had type 2 diabetes, and the mean diabetes duration was 20.5 ± 11.1 with a mean HbA1c of 67 ± 16 mmol/mol. 66/86 (76.8%) of patients had limb salvage, 7/86 (8.1%) had major amputation, and 13/86 (15.1%) died. Patients with positive PCT baseline values in comparison to those with normal values showed a lower rate of limb salvage (30.4 versus 93.6%, *p* = 0.0001), a higher rate of major amputation (13 versus 6.3%, *p* = 0.3), and a higher rate of hospital mortality (56.5 versus 0%, *p* < 0.0001). At the multivariate analysis of independent predictors found at univariate analysis, positive PCT was an independent predictor of major amputation [OR 3.3 (CI 95% 2.0-5.3), *p* = 0.0001] and mortality [OR 4.1 (CI 95% 2.2-8.3), *p* < 0.0001].

**Discussion:**

Positive PCT at admission increased the risk of major amputation and mortality in hospital patients with DFI and CLI.

## 1. Introduction

Diabetic foot infection (DFI) is a severe complication of diabetic foot ulcers (DFUs) which dramatically increases the risk of limb amputation and mortality [1].

Diagnosis of DFI is usually performed by clinical inspection, and the outcomes of patients with DFI are often related to concomitant comorbidities, mainly peripheral arterial disease [2].

Common inflammatory markers such as c-reactive protein (CRP), erythrocyte sedimentation rate (ESR), and white blood cells (WBCs) may be useful for monitoring the response to the treatment but may not specifically assess the severity of DFI and outcomes.

Procalcitonin (PCT) is a peptide precursor of the hormone calcitonin which is often undetectable or in very low concentrations (<0.05 ng/ml) in healthy people. In the case of infection, different tissues (kidney, adipose tissue, lung, and liver) secrete PCT and the blood concentrations can increase regardless of the underlying pathological condition [3].

Different studies have highlighted the role of PCT as a diagnostic marker for bacterial infection, which is often more effective than other common markers used in clinical practice, such as CRP, ESR, and WBC, and may be more reliable than some experimental markers such as interleukin-6 (IL-6) or interleukin-8 (IL-8) [4]. Furthermore, PCT is a prognostic marker of severity linked to mortality rates in infectious processes [5, 6].

Several observational studies have also found that PCT is a suitable marker to distinguish bacterial infections in DFUs [7–9]. Despite this fact, the prognostic role of PCT in DFI has never been clearly evaluated.

The aim of this study is to establish the prognostic role of PCT in hospital diabetic patients with critical limb ischemia (CLI) and DFI.

## 2. Materials and Methods

Consecutive inpatients with CLI and moderate-to-severe DFI who referred to our diabetic foot unit since October 2016 until September 2017 have been included.

Patients were treated by a preset limb salvage protocol including revascularization, wound debridement, antibiotic therapy, and offloading [10]. CLI was defined according to the combination of clinical findings and TcPO2 (<30 mmHg) or ankle-brachial index (<0.9) [11]. Revascularization was performed in all cases by endovascular approach. Moderate and severe infections were defined according to the Infectious Disease Society of America (IDSA) and International Working Group on Diabetic Foot (IWGDF) [12]. Moderate infection was identified according to the involvement of structures deeper than the skin and subcutaneous tissues and the presence of erythema of >2 cm but without signs of systemic inflammatory response. Severe infection was identified when the local infection as described above is associated with at least 2 signs of systemic inflammatory response: temperature of >38°C or <36°C, heart rate of >90 beats/min, respiratory rate of >20 breaths/min or PaCO_2_ of <32 mmHg, WBCs count of >2000 or <4000 cells/*μ*l, or 10% immature [band] forms. Early debridement was performed in the case of abscess, compartmental syndrome, wet gangrene, and necrotizing fasciitis to avoid the progression of the infectious process; otherwise, curative surgery was performed after revascularization. Antibiotic treatment started as empirical broad-spectrum therapy and was later driven by culture, if required. Close monitoring of renal function, glycemic levels, electrolyte balance, anemia, and pain was performed.

Demographic data and comorbidities have been reported. Hypertension was considered when blood pressure values were higher than 140/90 mmHg or there was a need for antihypertensive therapy; dyslipidemia was classified as LDL of >70 mg/dl or a need for statin therapy [10]; ischemic heart disease was considered in the case of previous coronary acute syndrome, coronary revascularization, or electrocardiogram abnormalities [13]; carotid artery disease was considered in the case of occlusion or stenosis of >50% or in the case of previous carotid revascularization. Dialysis was considered when chronic renal replacement therapy was required.

Inflammatory markers, including PCT, were evaluated for all patients at admission. Positive values of PCT were considered if greater than 0.5 ng/ml. Hospital outcomes were determined by limb salvage (discharge with preserved limb), major amputation (amputation above the ankle), and mortality.

Statistical analysis was performed by SAS (JMP12; SAS Institute, Cary, NC) for the personal computer. Data are expressed as means ± SD. Univariable logistic regression analyses were performed for all potential predictor variables with the outcome of interest (limb salvage, major amputation, and mortality) with values presented as univariable odds ratios (ORs) along with the respective 95% CI. Then, all potential predictors were entered simultaneously in a multivariate logistic regression model. These models yielded a set of variables that best predict the outcome. *p* < 0.05 was considered statistically significant.

## 3. Results

Eighty-six patients were included. Baseline characteristics are reported in Table 1.

### 3.1. Outcomes

66/86 (76.8%) of patients had limb salvage, 7/86 had (8.1%) major amputation, and 13/86 (15.1%) died.

6/10 patients died from sepsis, 4/10 from acute coronary syndrome, 1 from acute heart failure, and 2 from sudden death.

Twenty-three (23/86) (26.7%) subjects had positive PCT values with a mean value of 4.7 ± 1 versus 0.02 ± 0.01 ng/ml.

Among the subjects with positive PCT, 15/23 (65.2%) had moderate infection and 8/23 (34.8%) had severe infection according to IDSA classification. Furthermore, patients with positive PCT reported a higher rate of ESRD and heart failure in comparison to patients with normal values of PCT (Table 2).

**Figure 1:**
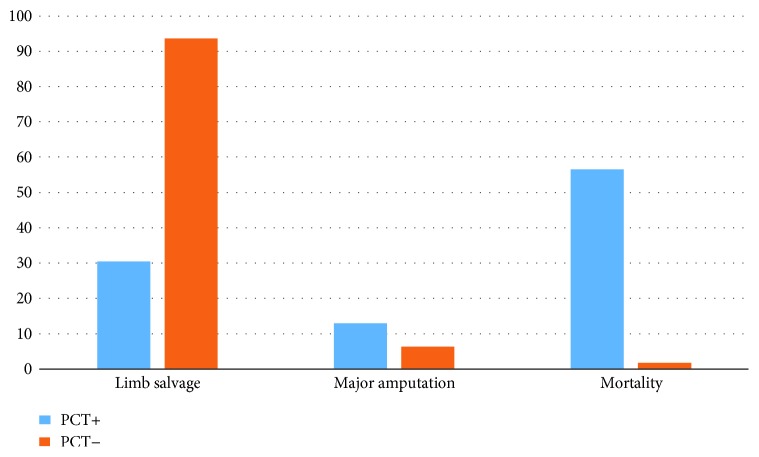
Hospital outcomes in patients with or without positive values of PCT. PCT: procalcitonin.

Patients with positive PCT baseline values in comparison to those with normal values showed a lower rate of limb salvage (30.4 versus 93.6%, *p* = 0.0001), higher rate of major amputation (13 versus 6.3%, *p* = 0.3), and a higher rate of hospital mortality (56.5 versus 0%, *p* < 0.0001) (Figure 1).

There were no significant differences in outcomes according to the values of other common inflammatory markers, including WBC, CRP, ESR, and fibrinogen, except for the higher values of CRP in the deceased patients in comparison with the survivors (Table 3).

### 3.2. Major Amputation

At the multivariate analysis of independent predictors found at univariate analysis, revascularization failure [OR 2.8 (CI 95% 1.9-3.1), *p* = 0.002] and positive PCT [3.3 (CI 95% 2.0-5.3), *p* = 0.0001] were independent predictors of in-hospital major amputation.

### 3.3. Mortality

At the multivariate analysis of independent predictors found at univariate analysis, positive PCT+ [OR 4.1 (CI 95% 2.2-8.3), *p* < 0.0001] and heart failure [1.8 (CI 95%1.05-5.5), *p* = 0.003] were independent predictors of in-hospital mortality.

WBC, ESR, CRP, and fibrinogen were not independent predictors of outcome.

## 4. Discussion

Although different papers have reported the usefulness of PCT as a diagnostic marker of DFI [9], only 2 studies have reported the association between PCT and amputation in DFI [14, 15]. This study is the first that evaluates the prognostic role of PCT in hospital patients with DFI and CLI.

In our study, PCT was only high in a small number of patients regardless of the severity of infection as based on IDSA guidelines. Nevertheless, patients with positive PCT at admission showed worse outcomes in terms of amputation and mortality in comparison to those with normal values.

Otherwise, the common inflammatory markers used in clinical practice such as WBC, CRP, ESR, and fibrinogen were not related to outcomes in this cohort of patients.

Furthermore, PCT was an independent predictor of amputation and mortality, together with failed revascularization and heart failure.

While revascularization failure is a known predictor of major amputation [14], the possible predictive role of PCT in lower limb amputation has already been investigated with conflicting results. In a small case-control pilot study with 27 patients, Karakas et al. did not find PCT levels to be significantly higher in amputees in comparison to non-amputees [15]. On the contrary, recently, in a larger case-control study of 156 patients with DFI requiring surgical intervention, Reiner et al. found that PCT values were significantly higher at admission (median 1.7 ng/ml) in patients who underwent below-the-knee or above-the-knee amputation, in comparison to patients whose limbs were salvaged (median 0.105 ng/ml) [16].

In our cohort of patients, 13% of subjects with positive PCT at admission underwent major amputation due to the progression of the infection and persistence of the limb ischemia after failed revascularization. Perhaps, positive PCT values may reflect an aggressive foot infection that is more difficult to treat in the presence of persistent poor blood perfusion.

PCT is a well-known prognostic factor of mortality, and it is currently used as marker of severity in infected patients, mainly in those with sepsis managed in acute care settings [5, 6, 17].

Heart failure is a documented predictor of mortality in patients with diabetic foot syndrome [18], while PCT has never been described as marker of mortality in patients with DFI and CLI.

Of the patients in this study, those with positive PCT values at admission had a very high rate of mortality (approximately 56%), and in the majority of cases, death was related to septic shock.

The patients described in this study are critically ill patients at the highest risk of adverse outcome: they were referred from the emergency department and they had several comorbidities and a small part already showed signs of sepsis at admission. It should be noted that not all patients with positive PCT were septic at the time of referral.

Additionally, PCT was high in some patients with moderate infection and in some patients with severe infection, regardless of the grade of the infection. Moreover, the grade of infection did not prove to be an independent predictor of outcome.

In relation to the main comorbidities among patients with positive procalcitonin, the rate of subjects on dialysis and with concomitant heart failure was extremely high, 56.5% and 52.2%, respectively. According to this data, it could be assumed that severe comorbidities such as renal and heart failure could predispose patients with infected DFUs to a systemic inflammatory response.

PCT may be considered a marker for a strong acute inflammatory response that reflects global deregulation, even in patients that are not ill with sepsis (i.e., patients with moderate DFI). Recent studies reported that the increase of PCT is not exclusively related to the infectious process, but there is a significant relationship between PCT and organ dysfunction and tissue injury as described after cardiac surgery in patients with acute myocardial ischemia [19, 20].

## 5. Conclusion

According to a review of the literature, this is the first study to evaluate the role of PCT as a marker of outcome in hospital patients with ischemic-infected DF.

According to our data, PCT may be considered as a predictor of mortality in hospital patients with CLI and moderate-to-severe infection and its prognostic role should be considered in the assessment of these patients, regardless of the clinical severity of infection.

It could be assumed that PCT may be useful for early diagnosis of systemic inflammatory response including nonseptic patients. It could help to identify high risk patients even without clear clinical signs and may be used to improve clinician's strategies (i.e., need of intensive unit, reinforcement of antibiotic therapy, and close monitoring of vital signs, hemodynamic parameters, and laboratory values). Nevertheless, it must be managed according to medical history and clinical evaluation.

### 5.1. Study Limitations

This is retrospective study and data has been collected from a single center. DFS is a complex disease, and it should be mentioned that other factors could influence final outcomes. Further studies are needed to evaluate if high values of PCT in patients with DFI could be related to concomitant comorbidities and if early analysis of PCT could improve outcomes.

## Figures and Tables

**Table 1:** Baseline characteristic of the whole population.

Variables	Whole population (*n* = 86)
Age (years)	67.3 ± 11.4
Sex (male)	80.7%
Diabetes (type 2)	95.1%
Diabetes duration (years)	20.5 ± 11.1
HbA1c (mmol/mol) (%)	67 ± 16 (8.3 ± 3.6)
Hypertension (yes)	73.1%
Dyslipidemia (yes)	72.1%
Current smokers	11.1%
IHD	72.8%
Heart failure	27.9%
CAD	15.8%
ESRD (dialysis)	37.2%
Procalcitonin (ng/ml)	1.3 ± 0.3
ESR (mm/h)	104 ± 14
CRP (mg/dl)	85.6 ± 47.8
WBC (×10^3^/ml)	11.3 ± 2.5
Fibrinogen (mg/ml)	455 ± 165
Wound parameters	
Dimension (>5 cm^2^)	83.1%
TcPO_2_ basal (mmHg)	22.4 ± 7.9

IHD: Ischemic heart disease; CAD: carotid artery disease; ESRD: end-stage renal disease; ESR: erythrocyte sedimentation rate; CRP: c-reactive protein; TcPO_2_: transcutaneous foot oximetry; WBC: white blood cells; PTA: percutaneous transluminal angioplasty.

**Table 2:** Positivity of procalcitonin according to the degree of infection and comorbidities.

Variables	Positive procalcitonin	Normal procalcitonin	*p* values
Moderate infection	15/23 (65.2%)	—	
Severe infection	8/23 (34.8%)	—	
ESRD	13/23 (56.5%)	19/63 (30.1%)	0.0002
IHD	17/23 (73.9%)	45/63 (71.4%)	0.2
Heart failure	12/23 (52.2%)	12/63 (19%)	0.0001

ESRD: End-stage renal disease; IHD: ischemic heart disease.

**Table 3:** Relation between baseline inflammatory markers and outcomes.

Inflammatory markers	Survival with MA	Survival without MA	*p*	Survivors	Deceased	*p*
WBC (×10^3^/ml)	10.9 ± 1.6	11.3 ± 2.4	0.1	11.2 ± 2.1	11.5 ± 1.8	0.2
CRP (mg/dl)	90 ± 27	87.7 ± 34	0.3	87.9 ± 33	100.8 ± 55	0.04
ESR (mm/h)	108 ± 17	104 ± 13	0.8	104 ± 14	110 ± 9	0.08
Fibrinogen	462 ± 190	452 ± 158	0.07	453 ± 164	466 ± 195	0.06

MA: Major amputation; WCB: white blood cells; CRP: c-reactive protein; ESR: erythrocyte sedimentation rate.

## Data Availability

The description of data used to support the findings of this study are included within the article.
